# Gabapentin-Induced Myoclonus in a Patient With Chronic Kidney Disease

**DOI:** 10.7759/cureus.47351

**Published:** 2023-10-19

**Authors:** Ammar N Mohamed, Marie-Alex Michel, Samy I McFarlane

**Affiliations:** 1 Internal Medicine, State University of New York Downstate Medical Center University Hospital, Brooklyn, USA; 2 Internal Medicine, Kings County Hospital Center, Brooklyn, USA; 3 Internal Medicine, Veterans Affairs Medical Center, Brooklyn, USA; 4 Nephrology, Veterans Affairs Medical Center, Brooklyn, USA; 5 Internal Medicine, State University of New York Downstate Health Sciences University, Brooklyn, USA

**Keywords:** renal dose adjustment, gabapentin neuro, action myoclonus-renal failure syndrome, severe diabetic neuropathy, painful neuropathy, ckd management, pain management in ckd patients, ckd, drug-induced myoclonus, pregabalin and gabapentin treatment for peripheral neuropathy

## Abstract

Gabapentin contains a cyclohexyl group and is a form of gamma-aminobutyric acid (GABA). Despite its name, gabapentin does not affect the inhibitory neurotransmitter GABA or its receptors. Instead, it acts as a ligand, binding strongly to the α2δ (Ca) channel subunit and interfering with its regulatory function and the release of excitatory neurotransmitters. Gabapentin is approved by the FDA for treating seizure disorders and neuropathic pain, except for trigeminal neuralgia. However, it is frequently used off-label to treat other pain conditions and psychological disorders, such as anxiety. Unlike other drugs, gabapentin is not metabolized in the liver and is solely excreted by the kidneys. Therefore, it is crucial to adjust the dosage in patients with renal insufficiency to avoid severe adverse effects. In this case report, we present a patient with chronic renal impairment who experienced devastating myoclonic jerky movements shortly after increasing his gabapentin dose.

## Introduction

Gabapentin is an anticonvulsant medication, commonly used to manage neuropathic pain, and it also finds widespread off-label use in treating various pain and sleep disorders. Notably, gabapentin is exclusively excreted through the kidneys, making its dose reduction essential when given to patients with impaired renal function. The appropriate dosing based on the patient’s actual creatinine clearance is imperative to prevent severe adverse side effects and drug-related toxicity. We report a case of myoclonic activity developed in a patient with chronic kidney disease (CKD) shortly after a gabapentin dose increase.

## Case presentation

An 83-year-old man with a past medical history significant for type 2 diabetes mellitus, hypertension, hyperlipidemia, stage 3b CKD, obstructive sleep apnea, coronary artery disease, benign prostatic hyperplasia, recent excision of a left posterior auricular mantle cell lymphoma, and neuropathic pain presented for evaluation of worsening involuntary upper and lower extremity movements and hiccups. The patient has been experiencing chronic neuropathic pain in his lower extremities for which he was on gabapentin 300 mg twice daily. The pain had worsened in the last few months which prompted his primary care provider to increase his gabapentin dose to 300 mg three times daily, with adequate pain control. Four days prior to hospitalization, the patient developed fast and repetitive jerking involuntary movements of all extremities (more prominent in his upper extremities), facial twitching, and hiccups. The movements were unintentional, rapidly progressive, and disabling, causing impairment in daily activities. The hiccups have worsened significantly during the last two days prior to hospitalization, making the patient unable to swallow even his daily medications (including gabapentin). No history of similar symptoms or seizures was reported, and he remained alert and oriented with no change in his mental status. His social history was negative for smoking, alcohol, or recreational drug use.

His medications include gabapentin, aspirin, allopurinol, empagliflozin, glipizide, hydrochlorothiazide, losartan, metoprolol succinate, and pantoprazole. His vitals include the following: temperature 97.9F (36.6 C), blood pressure 130/69 mmHg, pulse 78 bpm, weight 155.7 lb (70.62 kg), height 64 in (162.6 cm), BMI 26.8 kg/m2, and pain score 0/10.

His physical exam was significant for constant twitching of extraocular muscles and hyperkinetic repetitive jerky movements involving the upper and lower extremities. The movements were more prominent in the upper extremities. Facial twitching and diaphragmatic myoclonus were also noted. Additionally, the patient had persistent hiccups. The power and tone of all extremities were normal, and knee reflexes were 2+ (brisk response). The rest of the physical exam was unremarkable.

**Figure 1:**
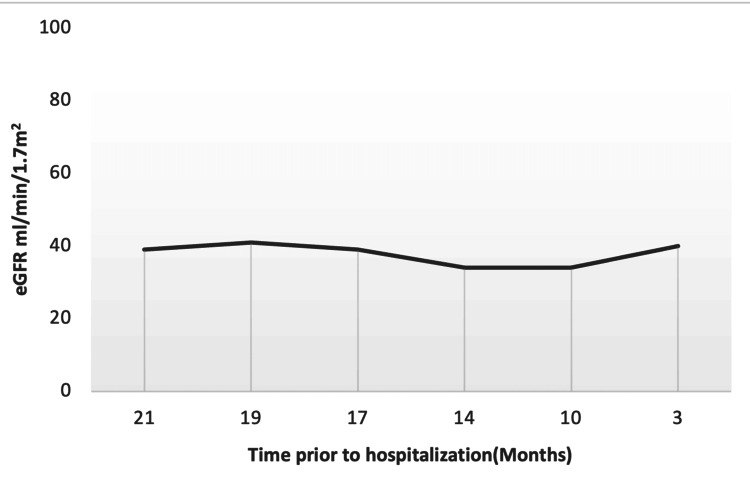
eGFR follow-up over time (months) prior to hospitalization eGFR: estimated glomerular filtration rate

Laboratory results on admission were significant for serum creatinine of 1.8 mg/dl, blood urea nitrogen (BUN) of 28 mg/dl, and estimated glomerular filtration rate (eGFR) of 37 ml/min/1.7m2 (Table 1 and Figure 1).

**Table 1:** Laboratory data Laboratory data over a period of 21 months, including the date of Hospitalization. BUN: blood urea nitrogen, Alk Po4: alkaline phosphatase, eGFR: estimated glomerular filtration rate, CO2: serum bicarbonate

Date	21 months prior to admission	19 months prior to admission	17 months prior to admission	14 months prior to admission	10 months prior to admission	3 months prior to admission	On admission	Reference range
BUN mg/dl	35	34	40	36	46	28	28	7-25
Creatinine mg/dl	1.8	1.7	1.9	2.0	2.0	1.7	1.8	0.7-1.3
Calcium mg/dl	8.6	9.1	8.9	8.2	9.1	8.7	9.1	8.2-10.0
Albumin g/dl	4.4	4.4	4.1	4.0	4.2	3.5	4.0	3.5-5.7
AlK Po4 U/L	104	104	94	108	113	74	98	34-104
CO2 mmol/l	22	22	22	24	24	22	25	21-31
eGFR ml/min/1.7m^2^	39	41	39	34	34	40	37	>=60

## Discussion

The kidneys solely excrete gabapentin with no hepatic clearance. In 2019, Kaufman et al. reported two unique cases of gabapentin-related toxicity and myoclonic activities. The first case was for a 78-year-old woman with congestive heart failure and diabetes with peripheral neuropathy who presented with upper extremity myoclonic activities with normal mental status in the setting of azotemia from over-diuresis. The patient was chronically treated with gabapentin 900 mg daily. The symptoms quickly improved with the discontinuation of gabapentin and two sessions of hemodialysis (HD). The second case was for a 55-year-old man with a history of end-stage renal disease (ESRD) on peritoneal dialysis (PD), diabetes mellitus, neuropathic pain, and peripheral vascular disease who was evaluated for acute worsening severe upper extremity tremor and altered mental status shortly after initiation of gabapentin 600 mg total daily dose for neuropathic pain. Gabapentin was discontinued and the patient's PD exchanges increased from four to six daily. His mental status returned to baseline, and the upper extremity tremors resolved completely with these measures [1].

In an updated meta-analysis of previously published data on the effectiveness of gabapentin in treating chronic neuropathic pain and fibromyalgia and its adverse events, the calculated number needed to treat was 50% pain reduction. The author concluded that gabapentin was effective in some patients with chronic neuropathic pain. However, over half of the studied population showed no worthwhile pain relief [2,3].

It's evident from a literature review of previously reported cases that elderly individuals are at a higher risk of developing gabapentin-induced myoclonic activity even among those with normal renal function [4].

Rapid jerking involuntarily movements referred to as negative myoclonus have been reported in patients on gabapentin even in those with normal renal function who received a considerably nontoxic dose shortly after the administration of the medication [5].

Reaching a certain therapeutic concentration of gabapentin is inevitable to achieve the desirable effect, which makes its usage more challenging especially in patients with impaired renal function given its 100% renal clearance. Gabapentin does not undergo any hepatic metabolism and it's excreted unchanged in the urine. In individuals with normal renal function, gabapentin clearance is 100 ml/min, which is equivalent to a normal creatinine clearance. On the other hand, in patients with ESRD and on HD, gabapentin clearance with HD is approximately 142 ml/min with an elimination half-life of about four hours with HD, which makes HD an effective treatment option for gabapentin toxicity in patients with ESRD [6].

In this case report, we initially faced challenges in obtaining medical history due to severe disabling generalized myoclonus with the involvement of the oropharyngeal muscle as evidenced by the repeated hiccups and diaphragmatic myoclonus. The patient was kept nil per oral and intravenous normal saline at 125 ml/hour was provided. Urine studies were negative for toxins. On admission, eGFR was 37 ml/min/1.7m2, which decreased from 40 ml/min/1.7m2 three months prior to hospitalization (Table 1 and Figure 1). One dose of 2 mg lorazepam was given with short-term partial improvement. A head CT scan showed no intracranial pathology. EEG was planned. However, it was not performed given the rapid improvement of his symptoms. The patient recovered within 24 hours of hospitalization, enabling us to obtain a detailed history of his symptoms. The only contributing factor was the recent increase of his gabapentin dose from 600 mg daily to 900 mg total daily dose for his worsening lower extremities neuropathic pain. The dose increase was still within the acceptable maximum dose adjustment for his GFR. However, the fluctuation and the recent drop in the eGFR increased his risk of gabapentin toxicity (Table 1).

The limitation of this case report is that the clinical symptoms were attributed to gabapentin without measuring the gabapentin plasma level, and no EEG was performed given his quick recovery and the patient's request not to perform further investigation and laboratory testing. It's recognized in the literature that gabapentin's toxic and therapeutic plasma levels vary between patients, making the measurement of its plasma level of limited value in diagnosing or excluding the diagnosis of gabapentin-induced adverse events [7]. The diagnosis, in this case, was supported by the direct association of the recent dose increase of gabapentin in a patient with stage 3b CKD with the recent worsening of his renal function and the improvement of his symptoms after discontinuation of the offending agent, keeping in mind that our patient was not able to take gabapentin for over 48 hours prior to admission due to his worsening hiccups and myoclonus.

## Conclusions

Myoclonus is a well-reported complication of gabapentin toxicity especially in patients with renal impairment. As gabapentin is solely excreted by the kidneys, renal dose adjustment is recommended in the literature. However, patients with CKD remain at risk of life-threatening neurotoxic adverse events even with appropriate dosing for GFR. The devastating adverse event reported in this case imposes caution when prescribing gabapentin, especially in patients with impaired renal function. In the past, the incidence of such an adverse event might be relatively low. However, more cases are expected to be reported given the worldwide rise in the off-label use of gabapentin in treating different pain disorders and other psychiatric conditions. Physicians must be aware of such an adverse event and abstain from the off-label use of gabapentin in patients with CKD and ESRD.
